# The SARS-Cov-2 Proliferation Blocked by a Novel and Potent Main Protease Inhibitor via Computer-aided Drug Design

**DOI:** 10.22037/ijpr.2021.114846.15061

**Published:** 2021

**Authors:** Sepideh Shayan, Shahab Jamaran, Rafee Habib Askandar, Arian Rahimi, Azam Elahi, Chiako Farshadfar, Noeman Ardalan

**Affiliations:** a *Department of Biochemistry, Faculty of Biological Sciences, North Tehran Branch, Islamic Azad University, Tehran, Iran. *; b *Department of Microbiology, Faculty of Biological Sciences, Arak Branch, Islamic Azad University, Arak, Iran. *; c *Research Center, Sulaimani Polytechnic University, Sulaimani, Iraq. *; d *Young Researchers and Elite Club, East Tehran Branch, Islamic Azad University, Tehran, Iran.*; e *Clinical Research Development Center, Imam Reza Hospital, Kermanshah University of Medical Sciences, Kermanshah, Iran.*; f *Department of Biochemistry, Faculty of Biological Sciences, Science and Research Branch, Islamic Azad University, Sanandaj, Iran.*; g *Department of Microbiology, Faculty of Biological Sciences, Science and Research Branch, Islamic Azad University, Tehran, Iran.*

**Keywords:** COVID-19, Protease inhibitors, ADMET, Molecular dynamic, PCA, DCCM, MM-PBSA

## Abstract

The recent prevalence of novel “*coronavirus* disease 2019” has expanded quickly globally, causing a universal pandemic. Herein, an effort was constructed to design a potent drug to inhibit the main protease of SARS-Cov-2 (3CLp) by means of structure-based drug design. A large library of the compounds was used for virtual screening. After molecular docking and ADME studies, we selected a compound with a better binding affinity to the 3CLp active site and acceptable ADME properties compared to the selected positive control drug. Molecular dynamic (MD) simulation (200 ns) and Molecular Mechanics–Poisson Boltzmann Surface Area (MM-PBSA) were used for further analysis. MD simulation outcomes have proved that the 3CLp-ZINC31157475 complex possesses a considerable value of dynamic properties such as flexibility, stability, compactness, and binding energy. Our MM-PBSA computation illustrates that ZINC31157475 is more potent (-88.03 kcal mol^-1^) than nelfinavir (-19.54 kcal mol^-1^) against COVID-19 3CLp.

Further, we have determined that the main residues of the 3CLp interact with ligands from per-residue binding energy. In conclusion, we suggest that ZINC31157475 can potentially treat COVID-19 by inhibition of the 3CLp. However, *in-vitro* and *in-vivo* study is essential for approval of this suggestion.

## Introduction

A novel pandemic was emerged in 2019 by severe acute respiratory syndrome *coronavirus* 2 (SARS-CoV-2); also, this novel pandemic called COVID-19 (1, 2). SARS-CoV-2 mortality is currently computed in the range of 0.5-6% (3). Data from previous studies have indicated that older patients, especially those with underlying disease, are at higher risk in the term of Covid-19 illness and death than younger persons (4). The SARS-CoV-2 virus has a higher transmission capacity than SARS-CoV in 2003. The sudden increase in confirmed cases has made it very difficult to control the spread of COVID-19 (5). In many countries, at least 25 drugs include chloroquine, azithromycin, hydroxychloroquine, favipiravir, lopinavir-ritonavir, ribavirin, nelfinavir, Thalidomide, remdesivir, convalescent plasma, steroids, interferon, and anti–IL-6 inhibitors have been investigated for a large number of COVID-19 patients (6-10). There is currently no clinical evidence to support the effects of drugs against COVID-19 (6). Therefore, efforts are still in progress to find the best therapeutic combination for this virus.

The large genome of the *coronavirus* is a single-stranded positive-sense RNA (11). The gene size is in the range of 26,000 to 32,000 base; the number of open reading frames (ORFs) of the SARS-Cov-2 genome varies from 6 to 11 (12). On January 10, 2020, the complete sequence of the SARS-Cov-2 genome was released, which helped the rapid detection of the virus in patients using reverse transcriptase-polymerase chain reaction (RT-PCR) (13). The SARS-Cov-2 genomic organization consists of a 5′-untranslated region (UTR), a replicase complex (ORF1ab), a membrane protein (M) gene, an envelope protein (E) gene, a nucleocapsid protein (N) gene, a spike protein (S) gene, 3′-UTR, and several non-structural ORF (14). The major protease required for the *coronavirus’s* proteolytic maturation is called M^pro^ (3-chymotrypsin-like protease (3CLp)). This enzyme is one of the attractive and best-characterized targets for drug design because it plays an essential role in the immune regulation and cleavage of virus polyproteins (15). 3CLp is accountable for cleavage of the eleven locations, resulting in 16 non‐structural proteins (NSP) in SARS-CoV-2 (16). The 3CLp (PDB ID: 6W63) was used as a drug target in the present study.

**Figure 1:**
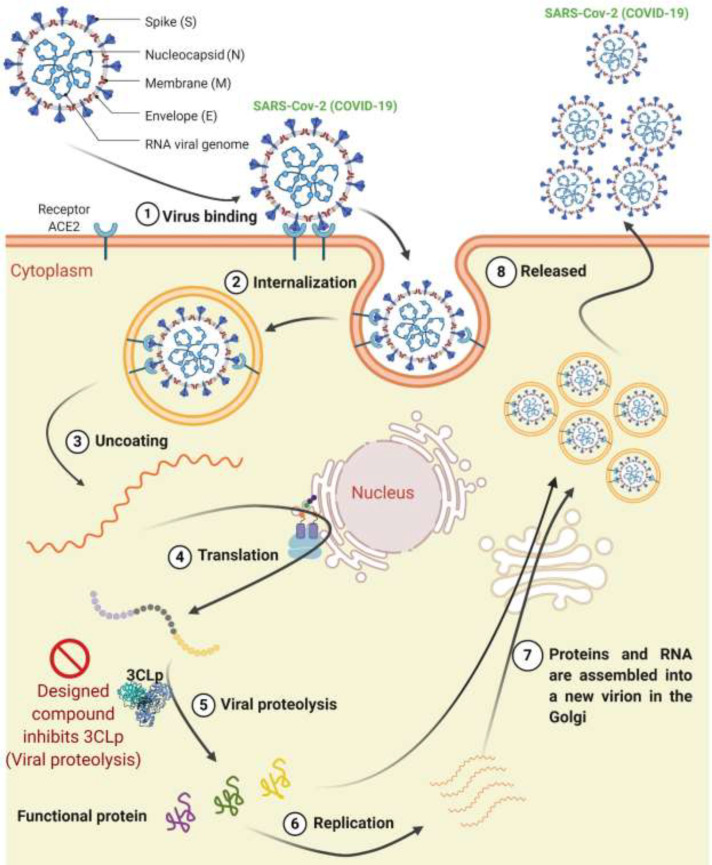
The life cycle of the SARS-Cov-2 in the cell consists of 8 steps. (1) Virus binding, (2) internalization, (3) Uncoating, (4) Translation, (5) Viral proteolysis, (6) Replication, (7) Assembling, and (8) Releasing. The designed compound inhibits 3CLp in the proteolysis step

The life cycle of the SARS-Cov-2 is as follows: The virus first attaches to the cell surface by binding to its receptor on the cell surface (ACE2) and enters the cell. After this step, the RNA is released, and a viral polyprotein is made inside the cytoplasm. This polyprotein is cleavaged by the 3CLp, and functional proteins are produced. The inhibitor designed in the present study inhibits this step of the virus life cycle (step 5 in Figure 1). Replication occurred after creating the viral protein, and the RNA and viral proteins are assembled into the new virion in the Golgi apparatus, and eventually, new virions release from the cell (Figure 1) (17, 18).

Drug discovery in the traditional procedure is a time-consuming process (more than a decade) (19), and finding an antiviral drug for COVID-19 in a short time seems to be essential. Hence, we cannot wait for the discovery of COVID-19 medicines through the traditional method. The rapid improvement of technology over the past few decades has opened many exciting ways to the scientist. One of them is computer-aided drug design (CADD), which utilizes computational software to assess the binding features of diverse compounds interacting with various targets (20). Therefore, CADD is an effective way to discover new drugs for diseases. CADD is essential to reduce laboratory costs and increase speed in drug discovery (21). The methods used in the present study include molecular docking, molecular dynamics (MD) simulations, principal component analysis (PCA), dynamical cross-correlation map (DCCM), and MM-PBSA.

Screening of approved drugs by researchers lead to recognized the HIV-1 protease inhibitor (nelfinavir) as a compound that prevented SARS-Cov-2 replication *in-vitro* (22). Previously, it has been demonstrated that nelfinavir is a potential inhibitor of SARS-Cov-2 protease (3CLp) based on CADD studies. Also, its antiviral activity was approved in duplicates in Vero E6 cells (23, 24). We have chosen nelfinavir as a positive control drug based on its docking energy.

## Experimental

*Ligands preparation*

We have used a large library of natural compounds provided from the ZINC database. This purchasable library by the name of AnalytiCon Discovery NP contains over 11200 compounds. The ligand structures were minimized, and then the necessary hydrogen was added by UCSF Chimera software (25).

*3CLp preparation and assign positive control*

The crystal structure of SARS-Cov-2 main protease (3CLp) (PDB ID: 6W63, Resolution: 2.10 Å, R-Value Free: 0.221, R-Value Work: 0.150) was engaged for molecular modeling simulations (26). Water molecules and heteroatoms, except native ligand (non-covalent inhibitor X77), were removed, and then bond orders were defined. Hydrogen atoms were added (whole non-polar hydrogen atoms were removed) to the structure. All-atom charges were calculated by UCSF Chimera using Gasteiger–Marsili method (25, 27, 28). To relax all closed contact and prevent clashes, the structure’s minimization was accomplished by the steepest descent method implemented in GROMACS 5.1 (29). Based on the previous studies, we have chosen several drugs for positive control includes remdesivir (30), chloroquine (31), hydroxyl-chloroquine (32), nelfinavir (11), ribavirin (33), arbidol (umifenovir) (34), dihydroergotamine (35), and glecaprevir (36).

*Docking study against 3CLp of SARS-Cov-2*

Molecular docking of the AnalytiCon Discovery library (11200 structures) into the active site of 3CLp was carried out by AutoDock Vina (37). This software is an academic-free molecular docking and uses a Python script collection (Autodock Tools (38)) to set up docking. AutoDock Vina works based on empirical scoring functions. All default parameters were set for docking runs (37). Finally, five compounds with the lowest docking energy were selected. The compound with the lowest docking energy was picked as a positive control for comparison with designed inhibitors among the studied control drugs. The 2D diagrams of compounds were created by web-based proteins plus software (https://proteins.plus/) (39).

*Computational ADME estimation*

An essential aspect of drug discovery is that avoided drugs do not have good absorption, distribution, metabolism, and excretion (ADME) properties (40, 41). Unfortunately, most of the drug candidates fail *in-vivo* studies due to poor ADME properties. Here, the ADME of the five best ligands and positive control were computed using the SwissADME. The 3D structure of chosen and positive control in mol2 format was imported to calculate the ADME properties. The SwissADME is a web-based software that predicts some drug features. SwissADME software can examine the physicochemical properties, lipophilicity, water-solubility, pharmacokinetics, drug-likeness, and medicinal chemistry (42). The absorption (ABS) was calculated using Equation 1 (43, 44). Finally, a ligand with the best feature was selected for MD simulation. 

ABS (%)= 109-[0.345× topological polar surface area (TPSA)]

 Equation 1. 

*Molecular dynamic (MD) simulation*

The best ligand and positive control selected after docking and ADME studies in complex with 3CLp of SARS-Cov-2 were introduced to MD simulation. All MD simulations were accomplished by GROMACS 5.1 collection with AMBER 99SB force field (29). The partial charges and topology files of ligands were computed by ACPYPE/AnteChamber (45). Each system was solvated with a TIP3P water model in a cubic box of 1 nm in periodic conditions, and related Cl^−^ and Na^+^ atoms were added to neutralize the system at a 0.15 mol L^-1^ concentration (46). Energy minimizations were done by the steepest descent and the conjugate gradient algorithm consecutively to achieve a maximum force of less than 1000 kJ mol^-1^ nm^-1^ on every atom. A twin-range cutoff scheme was determined to appraise short-range, non-bonded interactions, with van der Waals interactions truncated at 1.4 nm and electrostatic interactions truncated at 0.9 nm. To treat the Long-range electrostatic interactions, the particle mesh Ewald (PME) method was engaged (47, 48). The temperature was set at 300 K (ref_t) using velocity rescaling with a stochastic term and coupling time constant (tau_t) of 0.1 ps (49). This thermostat is comparable with Berendsen coupling, with the same scaling using tau_t, but the stochastic term ensures that a proper canonical ensemble is produced (29). The pressure was fixed at 1.0 atm using a Parrinello-Rahman barostat with a coupling constant of 2 ps (50). All of the covalent bonds involving hydrogen atoms were constrained by a Linear Constraint Solver (LINCS) algorithm to preserve constant bond lengths (51). The number of iterations to correct for rotational lengthening in LINCS (lincs_iter) and highest order in expanding the constraint coupling matrix (lincs_order) was assigned 1 and 4, respectively. Each system was equilibrated under a constant volume NVT (constant number of particles (N), volume (V), and temperature (T)) ensemble (100 ps) and a constant pressure NPT (constant number of particles (N), pressure (P), and temperature (T)) ensemble (100 ps) (52). All MD simulations were conducted for 200 ns. The trajectories were analyzed using free VMD software (53). The “readHBmap.py” Python script, was engaged to extract the percentage occupancy of H-bonds from H-bonds.xpm generated by h_bond GROMACS command (54).

*Principal component analysis (PCA)*

The PCA is a method that decreases the complexity of the data and draws out of the motion in simulations that are probably significant for biological function. In the PCA, a covariance matrix was manufactured from the trajectories after removing the rotational and translational movements. The computation of the projection of eigenvalues (Eigva) and eigenvectors (Eigve) along the first two PCA was performed using gmx_covar and gmx_anaeig GROMACS tools (55, 56). The Eigva and Eigve were distinguished by diagonalizing the matrix. Eigva demonstrates the amplitude of the Eigve along with multidimensional space, while the replacement of C_α_ atoms along each Eigve exhibits the concerted motions of the enzyme along each direction. This procedure divided the enzyme into two conformational subspaces. The first is an essential subspace, and the second is a physically non-essential subspace (57).

*Dynamical cross-correlation map (DCCM)*

To evaluation of the dynamics of systems, a DCCM was created to characterize cross-correlated displacements of the backbone C_α_ atoms during the simulations. DCCM demonstrated the largest motions within the system. The DCCM map was calculated by Equation 2 (58, 59):

$C_{\mathrm{ij}}=\frac{\left({\mathrm{\Delta r}}_{i}.{\mathrm{\Delta r}}_{j}\right)}{\left(\sqrt[{2}]{\langle {\Delta r}_{i}^{2}\rangle }.\sqrt[{2}]{\langle {\Delta r}_{j}^{2}\rangle }\right)}$

Equation 2.

Where i and j display i-th and j-th residues and 𝚫r_i_ and 𝚫r_j _correspond to the replacement of i-th and j-th atom from the mean position, respectively (58, 59), the DCCM map was created by an R base analysis tool (56, 60).

*Binding free energy computation*

Modified Molecular Mechanics–Poisson Boltzmann Surface Area (MM-PBSA) was engaged to compute binding free energy between ligands and receptors. Recently, the MM-PBSA has been used as a scoring function in silico drug design (61, 62). In the present study, the MM-PBSA method was carried out to estimate the interaction free energy between ligands and 3CLp of SARS-Cov-2. In our study, 2,000 snapshots were extracted at every 10 ps of intervals from the last 20 ns of MD trajectories. The free energy of binding is calculated by Equation 3:

G_binding_ = G_complex _– (G_protein_ + G_ligand_) 

Equation 3.

Where G_complex_ is the total free energy of the receptor-ligand complex, and G-protein and G_ligand_ are the total free energies of the alone protein and inhibitor in the solvent, respectively (63, 64).

## Results and Discussion

*Docking and ADME studies*

Five combinations with the lowest docking energy were selected among the 11200 natural products library (Table 1). The studied drugs were docked inside the active site of the 3CLp protease under the same condition. Among these drugs studied, remdesivir and nelfinavir have the best docking energy, but nelfinavir is a specialized protease inhibitor of human immunodeficiency viruses (HIV) (65) and remdesivir inhibits RNA-dependent RNA polymerase (66). Therefore, nelfinavir was selected as a positive control.

The ADME properties are listed in Table 2. SwissADME has computational filters that include Lipinski, Ghose (67), Ve­ber (68), Egan (69), Muegge rules (70). The ZINC31157475 has the best properties in these rules (Table 2). The ZINC31157475 has a molecular weight of fewer than 500 g-mol^-1^. Among all designed compounds, just the ZINC31157475 has a hydrogen bond (H-bond) acceptor lower than 10. All of the proposed natural products have a synthetic accessibility count of less than 10; thus, they are synthesized easily. The ZINC31157475 has the best value of synthetic accessibility. All of the proposed compounds have the proper count of predicted octanol/water partition coefficient (log P_o/w_), which is the classical descriptor for lipophilicity (valuable range of log P_o/w _is -0.4 to +5.6) (71, 72). The TPSA acceptable range is between 20 and 130 Å^2^. TPSA is critical in the prediction of absorption and brain access (72). All compounds except the ZINC31157475 have higher TPSA than the normal range. The ZINC31157475 (66.73%) calculated ABS is higher than all compounds listed in Table 2. The solubility of the ZINC31157475 is better than nelfinavir (log S values in the range 0 to -2 are soluble) (73). The ZINC31157475 demonstrated high gastrointestinal (GI) absorption as indicated by its high affinity for permeability glycoprotein (P-gp substrate) (Table 2). Therefore, we selected the ZINC31157475. The docking energy of the ZINC31157475 was -8.391 kcal mol^-1^. The docking energy of the selected positive control (nelfinavir) was -7.54 kcal mol^-1^. 3CLp residues interacting with ligands after docking were shown in Table 1. His41 and Glu166 residues has most interacted with the ligands (Table 1).

*Evaluation of the 2D interaction of selected compounds*

**Figure 2:**
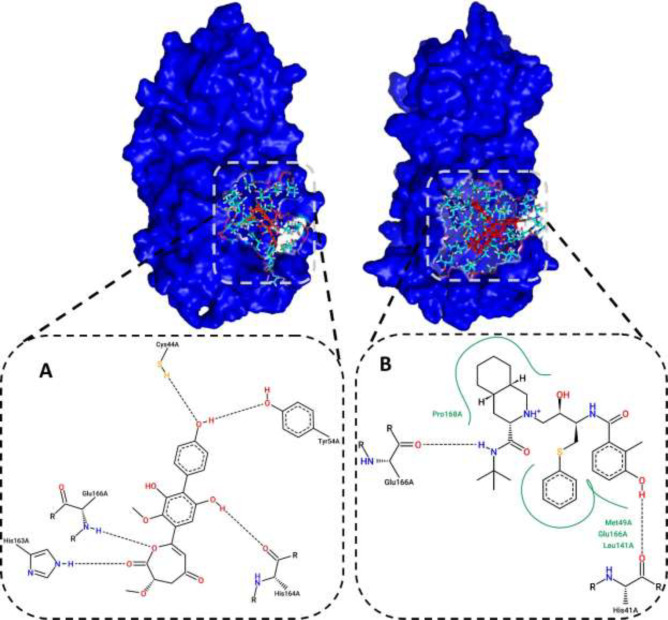
(A) 2D diagram of the ZINC31157475 and (B) nelfinavir at the 3CLp active site after docking. Hydrophobic interactions are shown in green, and H-bonds are shown in a black dashed line

Residues that interacted with the ZINC31157475 and nelfinavir are listed in Table 1. Here, we demonstrated the 2D diagram of these residues at the 3CLp active site (Figure 2). Five residues have interacted with a selected compound, including Cys44, Tyr54, His163, His164, and Glu166 (Figure 2A). There are five H-bond between these residues and the selected ligand. There is no hydrophobic interaction between the active site and the ZINC31157475. As seen in Figure 2B, His41, Met49, Leu141, Glu166, Pro168 residues have interacted with nelfinavir. There are fewer H-bond (two H-bonds) in the 2D diagram of nelfinavir than the ZINC31157475. H-bonds play a vital role in the attachment of ligands in the active site (74).

*Root-Mean-Square Deviation of 3CLp during the MD simulations*

**Figure 3:**
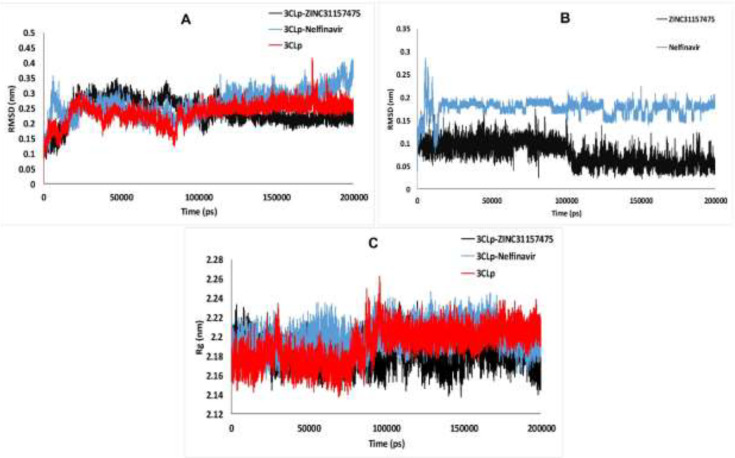
(A) Backbone RMSD, (B) Ligands RMSD, and (C) The computed radius of gyration of 3CLp-ZINC31157475, 3CLp-Nelfinavir, and 3CLp systems during 200 ns MD simulation

Root-Mean-Square Deviation (RMSD) of 3CLp-ZINC31157475, 3CLp-Nelfinavir, and un-ligated 3CLp systems during the MD simulations (200 ns) is shown in Figure 3. RMSD provides a better view of the stability of the studied system. RMSD of the un-ligated 3CLp (Red-line in Figure 3A) increases to 2.12 Å at the 3,180 ps. After that, RMSD was decreased to 1.23 Å at 10,650 ps. From 10,650 ps, an upward trend in RMSD starts and continues until 22,550 ps, at which time RMSD reaches 3.08 Å. Then, there is a downward trend until 38140 ps, which reaches 1.58 Å. In the range of 38,140-83,430 ps, the un-ligated 3CLp system reaches relative equilibrium. After this time, some drift is observed in the RMSD value, which finally reaches equilibrium after 100 ns of simulation (Figure 3A). The RMSD value of the 3CLp-Nelfinavir complex was reached 3.57 Å at 6,000 ps (Blue-line in Figure 3A). After that, an increase of RMSD value has happened until 11230 ps of the simulation. Then RMSD was increased to 3.08 Å at 26740 ps. A relative equilibrium was observed in the 3CLp-Nelfinavir system at 26,740-46,590 ps of MD simulation. The RMSD value was increased again to 3.29 Å at 48770 ps. Another drift has occurred, and the RMSD decreases to 1.72 Å at 53,110 ps. Then, slight changes in the RMSD value are observed till 100,000 ps. After this time, an upward trend in the value of RMSD is formed, which reaches 3.58 Å at 148,950 ps. Then at 160,840 ps, the RMSD value reaches 2.34 Å. Finally, the simulation was ended with an RMSD value of 3.58 Å at 200,000 ps (Figure 3A). The RMSD value of the 3CLp-ZINC31157475 complex (Black-line in Figure 3A) was increased to 3.25 Å at 22,630 ps. Then the system was reached equilibrium until 77,500 ps. After that, an increase of RMSD value was demonstrated to 3.3 Å at 79,640 ps of MD simulation. Then, after a slight decrease in the RMSD value, a proper equilibrium is observed until the end of the simulation (200 ns) (Figure 3A). The 3CLp-ZINC31157475 RMSD value is in the range of 0.80–3.45 Å, while 3CLp-nelfinavir is in the range of 0.82–4.06 for 200 ns simulation time. The average RMSD was 2.38 ± 0.4 Å (3CLp-ZINC31157475), 2.62 ± 0.43 Å (3CLp-Nelfinavir), and 2.35 ± 0.34 Å (un-ligated 3CLp). The 3CLp-ZINC31157475 RMSD value is lower than the 3CLp-Nelfinavir. Therefore, the 3CLp-ZINC31157475 was more stable than the 3CLp-Nelfinavir system (Figure 3A). 

The RMSD values of the ligands were also calculated and are shown in Figure 3B. Low fluctuations and low RMSD values (less than 2 Å) indicate high ligand stability in the active site cavity (75). The average RMSD value for ZINC31157475 and Nelfinavir was 0.76 ± 0.24 and 1.76 ± 0.17, respectively. Therefore, the ZINC31157475 inhibitor is more stable inside the active site.

*The radius of gyration analysis*

The radius of gyration (Rg) represents the folding and unfolding of protein structure during the MD simulation. Therefore, Rg was computed to distinguish the compactness of the system over the run time (Figure 3C). Higher Rg values elucidate less compactness, with high conformational entropy, while low Rg values show more stability and compactness in the structure (76, 77). The average Rg was 21.81 ± 0.13 Å (3CLp-ZINC31157475), 21.99 ± 0.13 Å (3CLp-Nelfinavir), and 21.92 ± 0.18 Å (un-ligated 3CLp). The data manifest that all three systems were compact and well converged throughout the simulation. The average Rg in the 3CLp-ZINC31157475 complex was less than the positive control system (3CLp-Nelfinavir), which indicates the binding of ZINC31157475 leads to increases in the stability and compactness of the enzyme compared to nelfinavir.

*Root-Mean-Square Fluctuation of 3CLp during the MD simulations*

**Figure 4:**
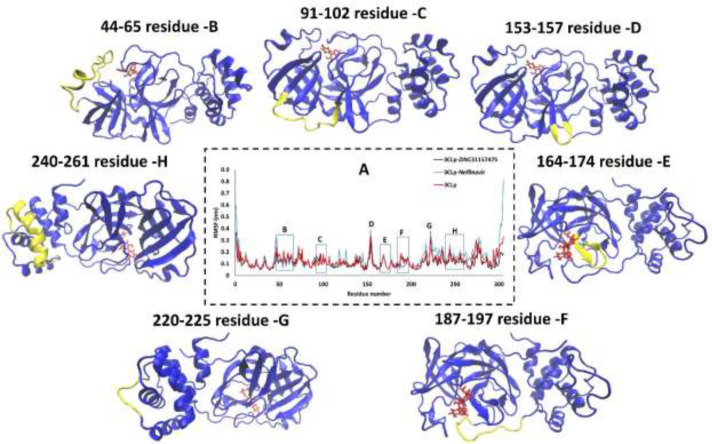
(A) RMSF of 3CLp-ZINC31157475, 3CLp-Nelfinavir, and 3CLp, 200 ns during the MD simulations, the residues 44-65 (B), residues 91-102 (C), residues 153-157 (D), residues 164-174 (E), residues 187-197 (F), residues 220-225 (G), and residues 240-261 (G) are shown in the 3CLp structure (Yellow).

Root-Mean-Square Fluctuation (RMSF) analysis versus the residue number for 3CLp-ZINC31157475, 3CLp-Nelfinavir, and un-ligated 3CLp systems during the 200 ns MD simulations were calculated by using the gmx_rmsf tool, and the results are illustrated in Figure (4). The RMSF of the backbone atoms is computed to decipher the flexibility of the structure. The high value of RMSF indicates the flexible region, while the low value of RMSF indicates limited motions during MD runs (78).

Three domains organize the 3CLp: domains I and II form the antiparallel *β*-barrel structure, while a compact α-helical domain is formed by domain III. The active site is located between domains I and II (79). Previous studies have shown that residues His41 (catalytic residue), Phe140, Asn142, Gly143, Ser144, Cys145 (catalytic residue), Tyr161, His163, Glu166, and His172 form the major part of the active site (80). The RMSF value of His41 (as a base catalyst) in the 3CLp-ZINC31157475 and 3CLp-Nelfinavir are 0.55 Å and 0.52 Å, respectively. The flexibility of the cys145 is lower (0.7 Å) in the term of ZINC31157475 binding than nelfinavir (1.92 Å) binding. It has been indicated that ZINC31157475 binding to Cys145 was better than nelfinavir.

There are seven regions in the RMSF plot (Figure 4A) in which the flexibility of the three systems is different. The first region (Figure 4B) includes 44-64 residues that residues 44-51 have lower flexibility after binding ZINC31157475 with 3CLp than nelfinavir binding. But residues 52-64 have higher RMSF in 3CLp-ZINC31157475 than 3CLp-Nelfinavir complex. There is a small helix in this region formed by 45-50 residue (81). The next region is the connecting loop (Loop C) between domains I and II (Figure 4C), which contribute to access to the active site (82). The flexibility of 3CLp-ZINC31157475 is higher than 3CLp-Nelfinavir in loop C (Figure 4A). The next loop (Loop D) is formed by residues 153-157 (Figure 4D), and in this small loop, the RMSF of 3CLp-ZINC31157475 is lower than 3CLp-Nelfinavir. The highest RMSF value in loop D is 2.91 Å and 3.8 Å for 3CLp-ZINC31157475 and 3CLp-Nelfinavir, respectively. The *β*-hairpin loop (Loop E) is prepared in Figure 4E. Loop E is formed by residues 166-170 (81). The *β*-hairpin loop consist of Glu166, and His172 residues, which are located in the active site. The RMSF value of the Glu166 and His172 in the 3CLp-ZINC31157475 complex is 0.75 Å and 0.74 Å, respectively. Besides, the RMSF value of the Glu166 and His172 in 3CLp-Nelfinavir is 1.06 Å and 0.75 Å, respectively. The lower RMSF value of the Glu166 in 3CLp-ZINC31157475 than 3CLp-Nelfinavir indicates a better binding of the ZINC31157475 to the active site (Figure 4). Loops are one of the vital structural parts of proteins. The roles of loops include specificity, regulating enzyme catalysis, stability, flexibility, and protein-protein interactions (83). The fifth region in Figure 4A is another loop that is formed by residues 187-197 (Loop F) (Figure 4F), which helps the ligands relocate to shift closer to the *β*-hairpin loop (81). The loop F is more flexible in the 3CLp-ZINC31157475 than 3CLp-Nelfinavir. More flexibility in loop F can be suitable because a high rate of fluctuations in loop F can conduct the ZINC31157475 inhibitor relocation to move closer to the *β*-hairpin loop. Region G in the RMSF plot illustrates the loop G with the most flexibility, in which residue 222 has an RMSF value of 3.72 Å and 3.62 Å in 3CLp-ZINC31157475 and 3CLp-Nelfinavir, respectively (Figure 4G). The final region is named H, which contains a helix and was formed by residues 240-260. In this residues the RMSF value of 3CLp-Nelfinavir is lower than 3CLp-ZINC31157475 (Figures 4A and 4H). Overall, the flexibility of 3CLp-ZINC31157475 is lower than 3CLp-Nelfinavir and un-ligated 3CLp.

*Hydrogen bond analysis*

**Figure 5:**
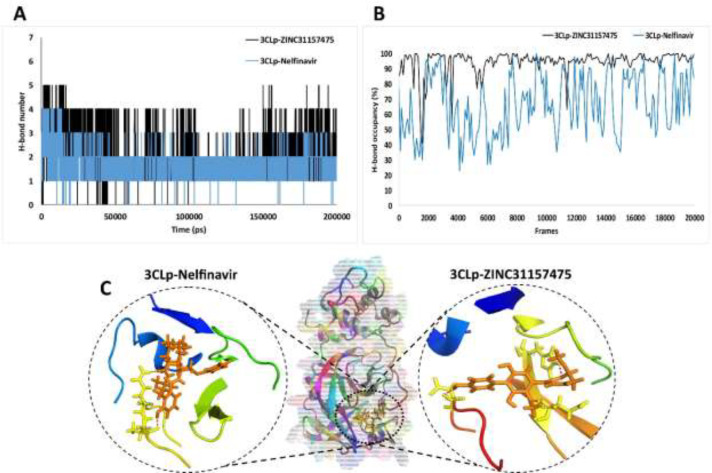
(A) The number of the hydrogen bond between 3CLp and ligands; 3CLp-ZINC31157475, 3CLp-Nelfinavir; (B) H-bonds occupancy (one frame equal 10 ps); (C) The 3D interaction between inhibitors and 3CLp active site, H-bond is shown in the red dashed line

H-bonds play a fundamental role in many protein features that include protein folding, the binding strength of protein-ligand interaction, and the catalysis function of the enzyme (84, 85). As we have shown in Figure 5A, the highest number of H-bond was four and five for 3CLp-Nelfinavir and 3CLp-ZINC31157475 complex, respectively. The results showed that in most of the simulation time, the number of H-bond in the 3CLp-ZINC31157475 was more than the 3CLp-Nelfinavir. The number of H-bond indicates the potent inhibitory of the ZINC31157475 compared to nelfinavir. The 3D interaction between drugs and the active site of the protein is examined after extracting a snapshot from the last frame of MD simulation (Figure 5C). The 3D interaction view is indicated that ZINC31157475 and nelfinavir make three and two H-bond with the active site residues, respectively. Also, there is a hydrophobic interaction between drugs and proteins (not shown in Figure 5C). On the other hand, there are a little more intermolecular H-bonds for 3CLp-ZINC31157475 when compared to 3CLp-Nelfinavir and un-ligated 3CLp (data not shown). More intermolecular H-bonds in the 3CLp-ZINC31157475 structure might make it more stable.

High H-bonds occupancy (the ratio of the number of times that particular H-bond is present relative to the total time of the simulation) indicates the stability of the H-bond during the MD simulation (86). The occupancy of H-bonds between 3CLp and ligands is shown in Figure 5B. Based on Figure 5B, the H-bonds formed between ZINC31157475 and 3CLp are more stable (higher occupancy) than nelfinavir H-bonds during MD time.

*Principal component analysis*

**Figure 6:**
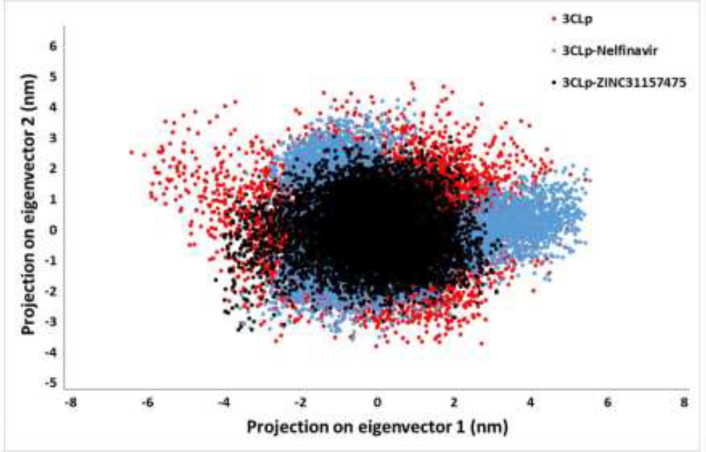
The principal component analysis (PCA) of 3CLp-ZINC31157475, 3CLp-Nelfinavir, and 3CLp. Projection of the motion of the un-ligated and ligated 3CLp in phase space along the eigenvector 1 and eigenvector 2

Overall, enzymes accomplish their specific roles through collective atomic motions. Hence, a collective atomic motion of a specific enzyme is employed as a parameter to figure out the stability of the enzyme (41, 87). The effect of the overall motion of enzymes due to ligands attachment was analyzed by PCA using the construction of eigenvectors. PCA is one of the powerful methods used to determine the rigidity of each atom and large-scale motions during the MD simulation (58). Figure 6 displayed the conformational sampling of un-ligated 3CLp and ligated 3CLp in the required subspace by projecting the C_α_ atom along eigenvectors 1 and 2. The results showed that 3CLp bound with ZINC31157475 had a different conformational fluctuation compared to nelfinavir binding (Figure 6). All three systems have good stability, but a reduction in the occupied conformational space by the 3CLp-ZINC31157475 complex is observed, which is consistent with the stability results of the RMSD, Rg, and the intermolecular H-bonds.

*Dynamic cross-correlation map*

**Figure 7:**
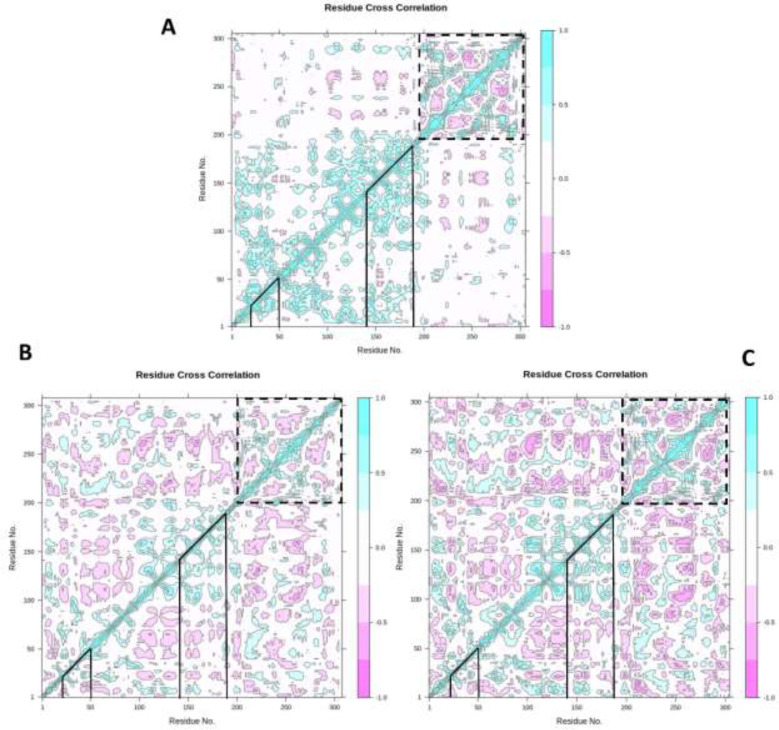
Dynamic cross-correlation map (DCCM) of un-ligated 3CLp (A), 3CLp-Nelfinavir (B), and 3CLp-ZINC31157475 (C). The value of correlated and anti-correlated motions is demonstrated based on color. The pink color indicates anti-correlated movements, and the cyan color indicates correlated movements. Deeper colors display stronger correlated and anti-correlated. The binding regions of the 3CLp are shown with lines, and domain III is shown with the dashed line

To illuminate the effect of ZINC31157475 and nelfinavir binding on the internal dynamics of 3CLp, the DCCM was constructed and is displayed in Figure 7. The DCCM analysis manifests the relevance between residues. Positive values (cyan color) illustrate residues that displace in the same direction, whiles negative values (pink color) are associated with the opposite displacement. The DCCM results indicated that ZINC31157475 and nelfinavir affect the structural remodeling of the 3CLp protease of SARS-Cov-2 as demonstrated by the change in the motions compared with the un-ligated 3CLp. Altogether, behind the ZINC31157475 and nelfinavir binding, a significant increase in the anti-correlated motions is seen for both complexes (Figure 7). The line determines the binding region of 3CLp in Figure 7A-C. The binding region included residues 20-50 and 140-190.

As seen in Figure 7, the un-ligated system showed an overall correlated motion at binding region residues highest than that of the ligated systems. There is not much difference in correlated motions in the binding region of the 3CLp-ZINC31157475 and 3CLp-Nelfinavir systems.‏ Both studied inhibitors increase anti-correlated motions in the binding region. Overall, the binding of ZINC31157475 and nelfinavir with 3CLp construct a stable environment around the binding cavity. In domain III (indicated by the dashed line in Figure 7), the anti-correlated motions are higher in domain III of the 3CLp-ZINC31157475 system. Overall, ZINC31157475 binding resulted in more anti-correlated motions (Deeper pink color) than nelfinavir in the protein; therefore, it indicates more stability of the 3CLp-ZINC31157475 complex.

*Binding free energy analysis*

Finally, at the end of the MD simulation, the binding free energy of inhibitors was calculated by MM-PBSA (Table 3). Based on these results, the binding free energy of nelfinavir and ZINC31157475 were -19.54 ± 37.80 kcal mol^-1^ and -88.03 ± 29.84 kcal mol^-1^, respectively (Table 3). Therefore, the 3CLp-ZINC31157475 complex has the lowest binding free energy. As shown in Table 3, only the polar solvation energy in the 3CLp-ZINC31157475 complex is higher than that of the 3CLp-Nelfinavir. The electrostatic energy of the 3CLp-ZINC31157475 complex (-61.61 kcal mol^-1^) is lower than the 3CLp-Nelfinavir complex (-39.19 kcal mol^-1^). The MM-PBSA results listed in Table 3, which manifest the improved interactions between the ZINC31157475 and 3CLp, give hope for a strong inhibitor.

**Figure 8:**
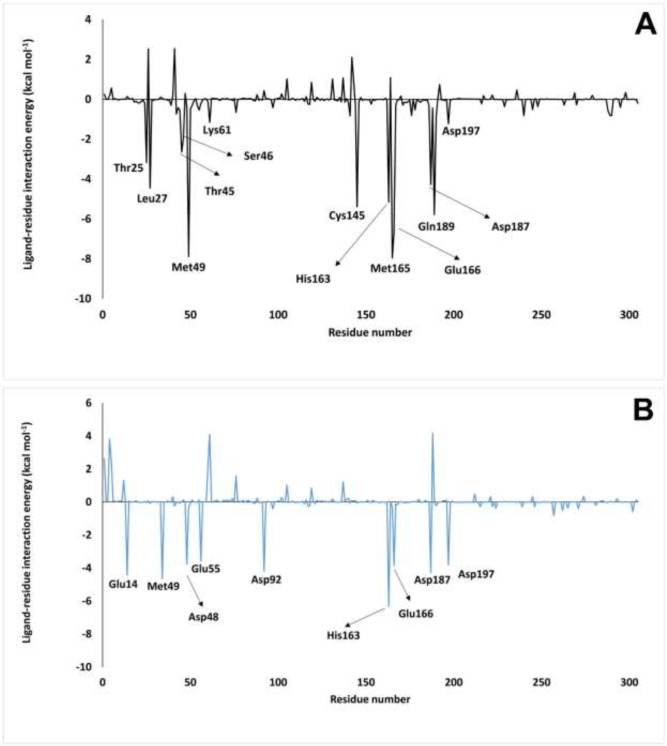
Per-residue ∆G_binding_ of 3CLp-ZINC31157475 (A) and 3CLp-Nelfinavir (B) complexes. The residue, which has lower than -1.0 kcal mol^-1^, is defined in the figure

Subsequently, we probed the vital residues engaged in the receptor-ligand binding by extracting the per-residue binding free energy using MM-PBSA. The per-residue binding free energy of both studied complexes was shown in Figure 8, and residues with lower energy than -1.0 kcal mol^-1^ are specified in the figure. The residues involved in the 3CLp-Nelfinavir binding include the Glu14, Met49, Asp48, Glu55, Asp92, His163, Glu166, Asp176, Asp187, and Asp197. Also, binding residues of the 3CLp-ZINC31157475 include the Thr25, Leu27, Thr45, Ser46, Met49, Lys61, Cys145, His163, Met165, Glu166, Asp187, Gln189, and Asp197. It is noteworthy that the designed inhibitor (ZINC31157475) binds to Cys145 (one of the catalytic residues) with appropriate energy (-5.39 kcal mol^-1^), but the binding energy of nelfinavir to His41 and Cys145 is -0.25 and -0.1 kcal mol^-1^, respectively.

**Table 1:** Summary of five top-ranked ligands and some drugs screened against SARS-Cov-2 3CLp with their ZINC ID, 2D structures, docking energy, and interacting residues

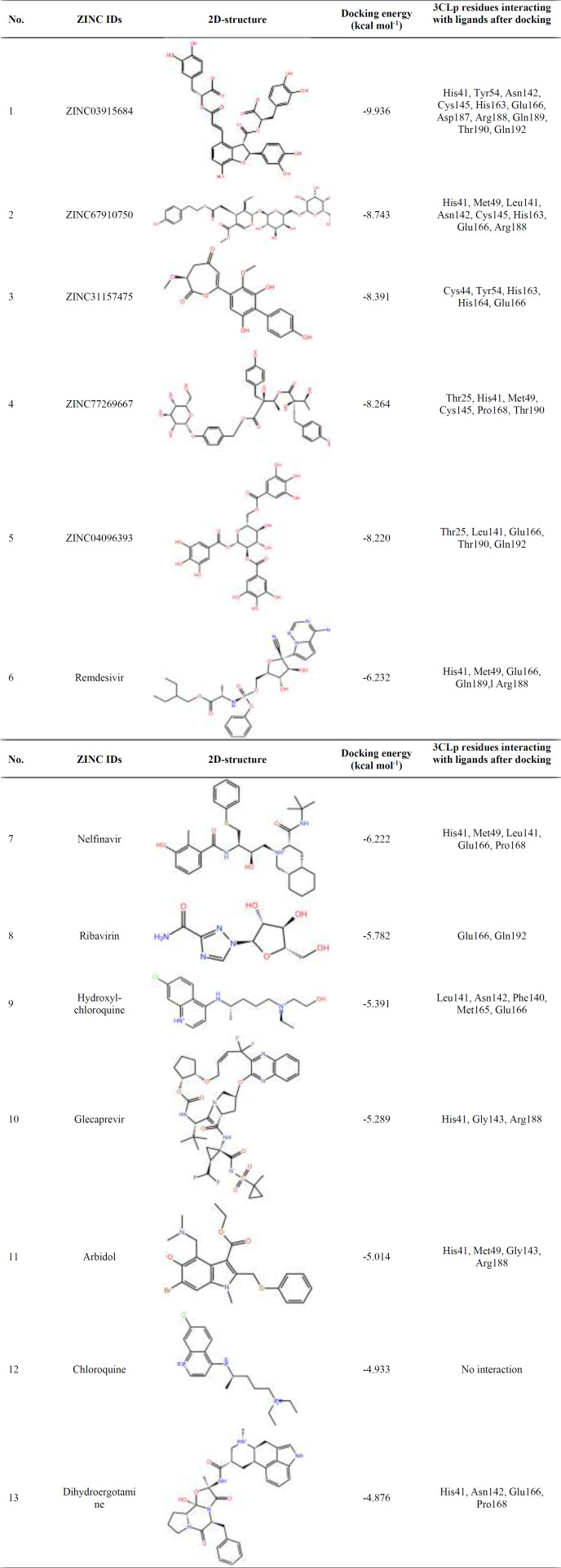

**Table 2:** SwissADME results, the selected compound showed in bold

**Properties**	**ZINC03915684**	**ZINC67910750**	**ZINC31157475**	**ZINC77269667**	**ZINC04096393**	**Nelfinavir**
MW^a^ (g mol^-1^)	718.61	686.65	386.35	702.70	636.47	567.78
Log S (ESOL) mol L^-1^	-6.22	-1.85	-3.44	-4.12	-3.65	-6.36
Solubility (mg ml^-1^)	6.02e-07	1.40e-02	3.67e-04	7.60e-05	2.24e-04	4.34e-07
Heavy atoms	52	48	28	50	45	40
No. H-bond acceptors	16	17	8	15	18	5
No. of rotational bonds	14	14	4	16	10	12
Log P_o/w_ (iLOGP)^c^	1.65	2.50	2.29	2.77	1.24	4.24
Lipinski’s rule of five (violation)	3	3	0	3	3	1
Ghose (violation)	3	4	0	4	2	3
Veber (violation)	2	2	0	2	1	1
Egan (violation)	1	1	0	1	1	0
Muegge (violation)	4	5	0	5	4	1
Bioavailability score	0.11	0.11	0.56	0.17	0.17	0.55
Synthetic accessibility	6.00	7.37	4.31	6.56	5.34	5.58
GI^d^ absorption	Low	Low	High	Low	Low	Low
BBB^e^ permeant	No	No	No	No	No	No
P-gp^f^ substrate	No	No	Yes	Yes	Yes	Yes
Log K_p_ (skin permeation) cm s^-1^	-7.86	-12.08	-7.36	-9.78	-9.93	-5.74
ABS %	13.07	19.09	66.73	21.67	1.82	65.11
TPSA (Å^2^)	278.04	260.59	122.52	253.13	310.66	127.20

^a^Molecular weight.

^b^Solubility.

^c^Predicted octanol/water partition coefficient.

^d^Gastrointestinal.

^e^Blood-Brain barrier.

^f^Permeability glycoprotein.

**Table 3:** The energy component of 3CLp-Nelfinavir and 3CLp-ZINC31157475 complexes was calculated by MM-PBSA

**3CLp- ZINC31157475**	**3CLp-Nelfinavir**	**Energy (kcal mol** ^-1^ **)**
-177.32 ± 58.11	-13.28 ± 42.88	∆E_vdw_^a^
-61.61 ± 21.36	-39.19 ± 40.22	∆E_elect _^b^
179.15 ± 52.88	38.32 ± 55.66	∆E_solv _^c^
-28.25 ± 6.25	-5.39 ± 5.38	∆E_SASA _^d^
-88.03 ± 29.84	-19.54 ± 37.80	∆G_binding_

^ a^van der Waal energy.

^ b^Electrostatic energy.

^ c^Polar solvation energy.

^ d^Solvent-accessible surface area energy.

## Conclusion

The SARS-Cov-2 has created a pandemic, so it is important to find effective drugs against it. We also conducted this study to design a potent drug against the main protease of this virus. We proposed the ZINC31157475 (natural product) as a SARS-Cov-2 protease (3CLp) inhibitor. The ZINC31157475 binds to the active site of the 3Clp with better docking energy (-10.463 kcal mol^-1^) than several control drugs includes remdesivir, chloroquine, hydroxyl-chloroquine, maraviroc, nelfinavir, ribavirin, umifenovir, dihydroergotamine, and glecaprevir. The RMSD value in the designed inhibitor complex is lower than the nelfinavir bonded complex. MD data revealed that the Rg is decreased in the presence of ZINC31157475 at the 3CLp active cavity. This decrement can be the result of the increase in H-bonds in the protein after ZINC31157475 binding. The RMSF decreasing has been observed after ZINC31157475 binding in comparison with unbound 3CLp protein and 3CLp-Nelfinavir complex. The atomic motions and internal dynamics were evaluated by PCA and DCCM methods, respectively. Based on the PCA data, the three (especially 3CLp-ZINC31157475) systems have admissible stability. DCCM analysis showed that the ZINC31157475 and nelfinavir binding lead to increment in the anti-correlated motions. Increasing anti-correlated motions generate a stable environment in the active cavity. We defined the residues that have high free binding energy to the ZINC31157475 ligand. Finally, binding free energy calculated by MM-PBSA showed that ZINC31157475 has a high potential for inhibition of the 3CLp activity. However, further laboratory and clinical trials are essential to evaluate the proposed therapeutic application.

## Conflicts of interest

The authors declare that they have no known competing financial interests or personal relationships that could have appeared to influence the work reported in this paper.

## Authors’ contributions

 Concept – Sepideh Shayan.; Design– Shahab Jamaran., Rafee Habib Askandar; Materials – Chiako Farshadfar., Noeman Ardalan.; Data Collection – Chiako Farshadfar., Noeman Ardalan.; Analysis– Sepideh Shayan., Noeman Ardalan., Shahab Jamaran.; PCA and DCCM analysis – Arian Rahimi., Azam Elahi.; Literature Search – Sepideh Shayan.; Writing – Noeman Ardalan., Sepideh Shayan.; Critical Reviews – Rafee Habib Askandar., Arian Rahimi., Noeman Ardalan., Azam Elahi., Arian Rahimi., Sepideh Shayan., Chiako Farshadfar. All authors have approved the final manuscript.
